# An Investigation of RSN Frequency Spectra Using Ultra-Fast Generalized Inverse Imaging

**DOI:** 10.3389/fnhum.2013.00156

**Published:** 2013-04-23

**Authors:** Rasim Boyacioglu, Christian F. Beckmann, Markus Barth

**Affiliations:** ^1^Radboud University Nijmegen, Donders Institute for Brain, Cognition and BehaviourNijmegen, Netherlands; ^2^MIRA Institute for Biomedical Technology and Technical Medicine, University of TwenteEnschede, Netherlands; ^3^Erwin L. Hahn Institute for Magnetic Resonance Imaging, University Duisburg-EssenEssen, Germany

**Keywords:** GIN, resting state, respiration, dual regression, ICA, frequency analysis, fMRI BOLD, physiological noise

## Abstract

With the advancements in MRI hardware, pulse sequences and reconstruction techniques, many low TR sequences are becoming more and more popular within the functional MRI (fMRI) community. In this study, we have investigated the spectral characteristics of resting state networks (RSNs) with a newly introduced ultra fast fMRI technique, called generalized inverse imaging (GIN). The high temporal resolution of GIN (TR = 50 ms) enables to sample cardiac signals without aliasing into a separate frequency band from the BOLD fluctuations. Respiration related signal changes are, on the other hand, removed from the data without the need for external physiological recordings. We have observed that the variance over the subjects is higher than the variance over RSNs.

## Introduction

Functional MRI (fMRI) studies related to “resting” brain has been one of the constantly growing fields in cognitive neuroscience (Biswal et al., 1995; Beckmann et al., 2005; Damoiseaux et al., 2006; De Luca et al., 2006; Smith et al., 2009; Laird et al., 2011). For the interpretation of resting state networks (RSNs) a clean mapping of relevant frequencies is of relevance to prevent the conclusion that RSNs are a mere artifact of physiological signals. Moreover, it has also recently been shown that RSNs are detectable for frequencies well above 0.1 Hz (Niazy et al., 2011; Smith et al., 2012; van Oort et al., 2012). It is therefore important that the main physiological fluctuations are sampled without aliasing into functionally relevant frequency bands. The breathing frequency is particularly problematic as the related frequency band is close to the main frequencies commonly associated with RSNs and as breathing leads to a more global effect than cardiac noise. For example, the default mode network, a commonly observed RSN, has been linked to respiration depth (Birn, 2012). The cardiac noise, however, is spatially localized to big vessels and arteries and introduces variance especially into the auditory network (Beall and Lowe, 2007).

Recent developments in MR acquisition techniques in 2D which are called simultaneous multislice imaging (SMS) enable sufficiently fast sampling of the MR signal to separate and remove the respiration related fluctuations (Moeller et al., 2009; Feinberg and Yacoub, 2012; Setsompop et al., 2012). However, if one aims to discern the cardiac noise, then ultra-fast MRI techniques, such as MR-encephalography (MREG) (Hennig, 2012; Zahneisen et al., 2012) or inverse imaging based methods (Boyacioglu and Barth, 2012b; Lin et al., 2012) should be the method of choice. Among these methods resting state analysis has been carried out with MREG by using a seed based correlation analysis (Lee et al., 2012) and with SMS by applying ICA (Feinberg et al., 2010). Both of these studies are proof of principle studies showing the benefits of increased time points and investigate the spatial characteristics of RSNs. Another study that used SMS dissected the RSNs into temporal functional modes (TFMs) by using temporal ICA (Smith et al., 2012). In this study we investigated the frequency spectra of RSNs by largely avoiding physiological contamination which could obscure functional interpretation. Therefore, we used a recently developed ultra-fast acquisition technique, generalized inverse imaging (GIN) (Boyacioglu and Barth, 2012b), with implicit acquisition of a phase regressor that resembles physiological fluctuations.

## Materials and Methods

### Data acquisition

The data were acquired with a 3-T MRI scanner (TIM Trio; Siemens Healthcare, Erlangen, Germany) and a 32-channel head coil. Six healthy subjects (one female, five male; aged 28–37) were recruited for the study and written informed consent was obtained according to the guidelines of the local IRB. Ultra-fast fMRI was performed using the GIN method (Boyacioglu and Barth, 2012b): the reference scan for the GIN reconstruction was carried out with a 3D EPI scan with the following parameters: in plane resolution 3.5 mm × 3.5 mm, slice thickness 3.5 mm, flip angle = 15°, TE/TR = 28/50 ms, 44 partition phase encoding steps, sagittal slices, FOV = 224 mm × 224 mm × 156 mm. The GIN data were acquired with the same parameters but with a 2D EPI scan with a slice thickness of 156 mm which resulted in a single collapsed slice in the left-right direction. Then, this slice was unaliased into a 3D volume using the GIN reconstruction framework. GIN uses the phase as a constraint to improve the solution of the highly undersampled regularized reconstruction and only needs a single 3D EPI prescan to obtain the necessary coil sensitivity information and reference images that are used to reconstruct standard images, so that standard analysis methods such as ICA and general linear model (GLM) are applicable. 5 min of resting state data (eyes open) were collected from each subject.

### Analysis

The data was preprocessed with FSL’s FEAT (v4.1.7)^1^ by removing the temporal drift and spatially smoothing with an 8-mm kernel. We have used DRIFTER (Särkkä et al., 2012) for physiological noise correction. It was shown that the phase drift time course, a by-product obtained during the GIN reconstruction, fluctuates with the respiration (Boyacioglu and Barth, 2012a) and was therefore used as the reference signal for DRIFTER to estimate the frequencies which were removed from the data. We have registered the eight template RSNs from Beckmann et al. (2005)^2^, to the individual subjects’ native space. Dual regression of the subjects’ functional data against these eight maps then gave rise to subject-dependent versions of these RSNs (Filippini et al., 2009). Dual regression analysis simply consists of two GLMs where the first one extracts the associated time course from the single subject data by using one of the RSNs as a spatial regressor and the second one uses that time course as a regressor to map the RSN onto the single subject level. The DICE overlap score (for subject *m* = 1, 2 … 6 and RSN *k* = 1, 2 … 8) was calculated to depict the similarity between a template (rsn*_k_*) and an individual subject (sub*_mk_*) map as follows,

(1) $$\text{dic}{\text{e}}_{mk}=\frac{2\left|\text{rs}{\text{n}}_{k}\cap \text{su}{\text{b}}_{mk}\right|}{\left|\text{rs}{\text{n}}_{k}\right|+\left|\text{su}{\text{b}}_{mk}\right|}$$

where || represents the number of voxels of a map and ∩ represents the intersection of two maps. Each RSN map on the single subject level was masked with a gray matter (GM) mask. The frequency spectra were normalized by their total power.

## Results

**Figure 1:**
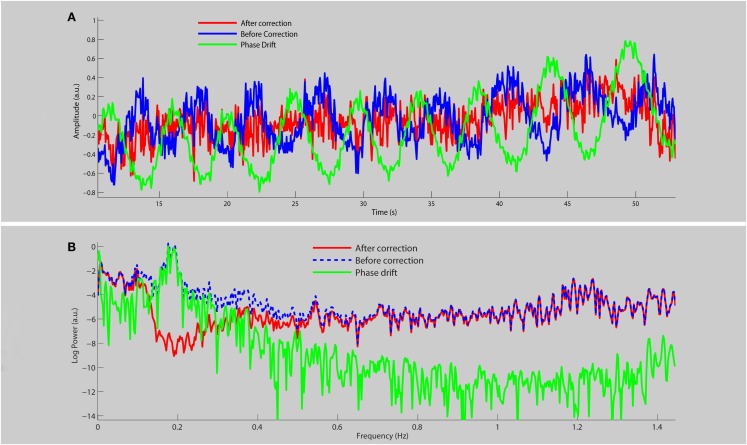
**A section of the average time course (A) and frequency spectrum on a log-linear scale (B) of RSNs for a single subject before (blue line) and after (red line) physiological noise removal, as well as the corresponding phase drift regressor (green line)**.

Figure 1 shows the average time course (a) and frequency spectra (b) for eight RSNs for a single subject before and after physiological noise removal. When the phase drift is used as the reference signal for physiological noise estimation/removal with DRIFTER, the data shows clear power reduction in the frequency range of breathing (see red line shown in Figure 1B), but the power at other frequencies is preserved and is free from respiratory fluctuations. Note that the phase drift time course carries little information about the cardiac signal (for this specific run around the principal cardiac frequency at 1.2 Hz) and thus does not reduce the power in that specific frequency band (see red line).

**Figure 2:**
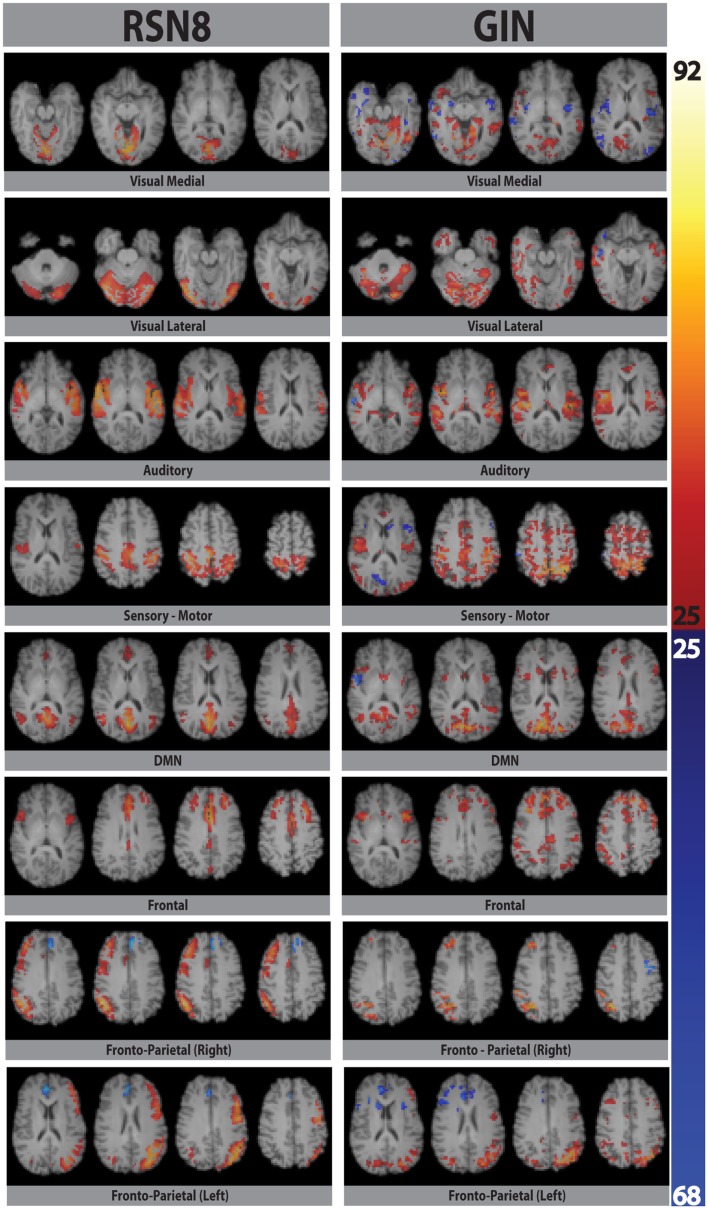
**Eight prototypical RSNs (Beckmann et al., 2005) and the corresponding dual regression maps (in *z*-scores) overlaid on the four most representative slices for a single subject**.

**Figure 3:**
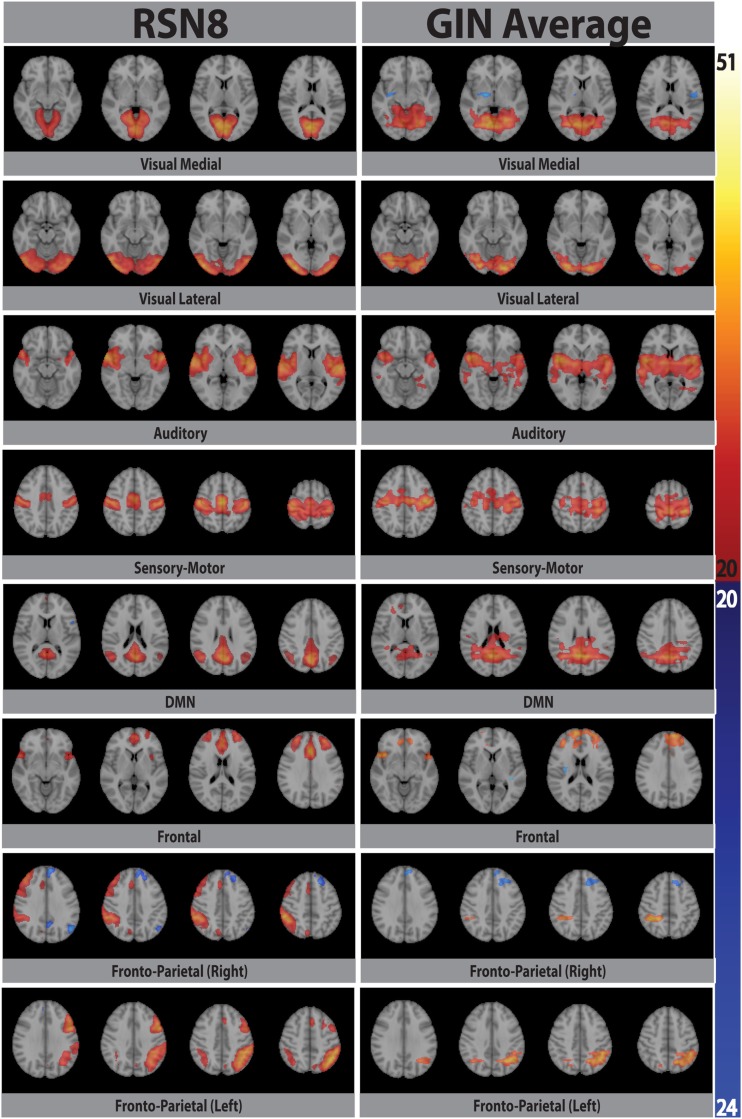
**Eight prototypical RSNs (Beckmann et al., 2005) and the corresponding dual regression maps (in *z*-scores) averaged over all subjects and overlaid on the four most representative slices in MNI space**.

Eight typical RSNs and the corresponding dual regression maps are shown in Figure 2 for a single subject. The spatial patterns of the prototypical RSNs are matched by their GIN counterparts. The similarity and spatial overlap between the dual regression maps and the typical RSNs is quantified with the DICE overlap score for all the subjects and RSNs in Table 1. The group level average of dual regression maps is shown in Figure 3. As the effective spatial resolution of GIN in the left-right direction is considerably reduced as a tribute to the high temporal resolution compared to fully encoded acquisitions the RSN maps are typically larger.

**Table 1:** **DICE overlap scores for all the subjects and RSNs**.

	Visualmedial	Visuallateral	Auditory	Sensorymotor	DMN	Frontal	Fronto parietal (right)	Fronto parietal (left)	Mean ± SD
S1	0.25	0.44	0.40	0.36	0.44	0.52	0.33	0.43	0.39 ± 0.08
S2	0.34	0.39	0.49	0.14	0.39	0.33	0.30	0.29	0.33 ± 0.10
S3	0.30	0.37	0.42	0.37	0.36	0.52	0.43	0.44	0.40 ± 0.07
S4	0.29	0.33	0.40	0.34	0.42	0.54	0.45	0.43	0.40 ± 0.08
S5	0.29	0.36	0.47	0.43	0.39	0.53	0.44	0.43	0.42 ± 0.07
S6	0.28	0.33	0.46	0.39	0.33	0.55	0.38	0.38	0.39 ± 0.08

**Figure 4:**
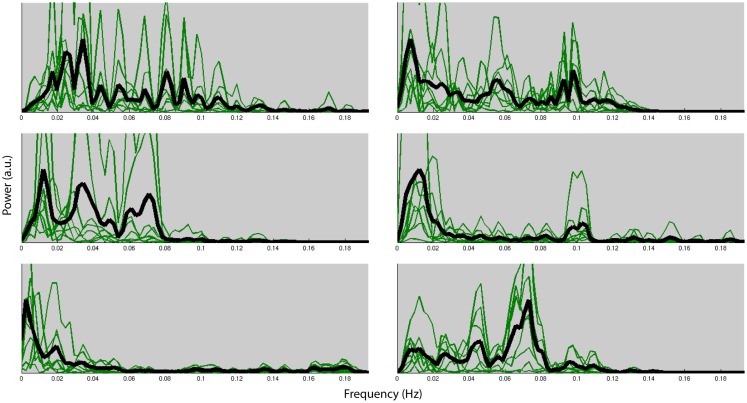
**Normalized frequency spectra of all RSNs (in green) and their average (in black) for each of the six subjects up to 0.2 Hz**. Variation over subjects is much higher than the variation over RSNs.

**Figure 5:**
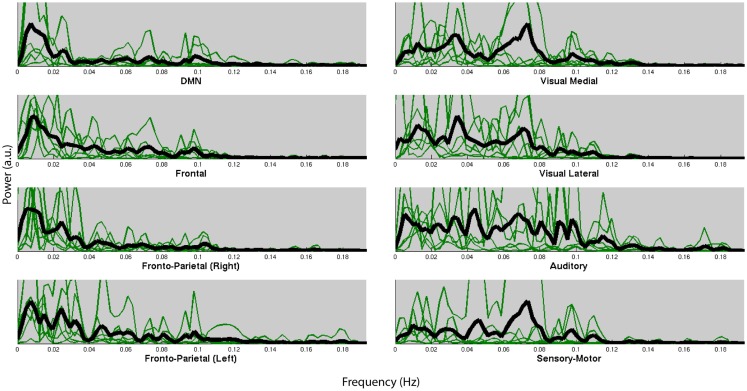
**Normalized frequency spectra of all RSNs (in green) and their average over subjects (in black) for each of the 8 RSNs up to 0.2 Hz**. RSNs do not have specific frequencies associated with all the subjects.

Figure 4 shows the normalized frequency spectra of all RSNs (in green) and their averages (in black) for all the subjects below 0.2 Hz. Most of the RSNs’ power reduces significantly above 0.1 Hz and the RSNs have similar frequency spectra within subjects. However, there’s considerable variation between the subjects’ average frequency spectra. Figure 5, on the other hand, shows each RSNs’ frequency spectra plotted for each subject (in green) and averaged over subjects (in black).

## Discussion

One very important point for using fast sampling in fMRI is that the main physiological fluctuations are sampled without aliasing into functional relevant frequency bands. For the interpretation of RSNs the breathing frequency is particularly problematic as the related frequency band is close to those frequencies commonly associated with RSNs. This has stirred some discussion to whether RSNs are an artifact of physiological signals (Birn et al., 2008; Birn, 2012). By using GIN, we are not only able to acquire the data fast enough but we can also correct for respiratory fluctuations, mostly due to bulk susceptibility changes, by using the information derived from the data itself. This can be seen from the blue curve in Figure 1B where respiration related signal changes are located in the frequency band of 0.15–0.25 Hz and are not only not aliased into lower frequencies, but also corrected for (red line). Since the phase drift time course is dominated by the global respiration signal, it matches the data within the same frequency band.

The phase drift time course does not carry much information about the cardiac signal (so very little variation around the principal cardiac frequency is removed), but this is of less concern as the frequency band is far from the typical RSN frequencies. Cardiac signals are much smaller in magnitude compared to respiration since they are localized to specific regions whereas respiration is a more global effect.

Within each subject we found very similar frequency characteristics for all RSNs, and that the variation over subjects was much higher than the variation over RSNs. Similar results have been reported in the literature (Niazy et al., 2011). These results could very likely be due to the result of differences of the hemodynamic response function (HRF) which is known to have high power in these frequencies (<0.1 Hz).

While the spatial fidelity of RSNs was not the specific focus of this study due to the inherent lower spatial resolution of GIN, all RSNs were spatially matched by their dual regression GIN counterparts, some (DMN, frontal) better than the others (fronto-parietal right, visual), however both the fronto-parietal networks – including their associated anti-correlated clusters – are recovered with GIN resting state data. As GIN does not have any gradient encoding in the left-right direction but uses the coil sensitivity information to separate the aliased voxels, this inevitably results in the trade off spatial resolution for increased temporal resolution. The effective resolution depends on the independent and uncoupled information available from the coil channels. In general, the effective resolution is higher for GM than white matter and poses fewer problems for fMRI. Naturally, the lower spatial resolution of GIN leads to a larger spatial extent especially in the left-right direction for some of the networks, leading to some of the relatively low DICE scores in Table 1. Another drawback of low TR acquisitions and GIN is the abundance of physiological noise related components obtained with regular ICA as they dominate the total variance in the data. The dual regression approach used in the study enabled to directly obtain network specific frequency spectra and overcome the disadvantages of GIN and low TR acquisitions.

Studies related to temporal ICA (Smith et al., 2012) and the high frequency content of RSNs (Niazy et al., 2011; van Oort et al., 2012) would certainly benefit from the large number of time points obtained with GIN in relatively short scan times.

## Conflict of Interest Statement

The authors declare that the research was conducted in the absence of any commercial or financial relationships that could be construed as a potential conflict of interest.

## Footnotes

^1^http://www.fmrib.ox.ac.uk/fsl/

^2^http://www.fmrib.ox.ac.uk/analysis/royalsoc8/
